# Acute Myocardial Infarction after First Dose of Rituximab Infusion

**DOI:** 10.4274/Tjh.2013.0247

**Published:** 2014-03-05

**Authors:** Ajay Gogia, Sachin Khurana, Raja Paramanik

**Affiliations:** 1 B. R. A. Institute Rotary Cancer Hospital, All India Institute of Medical Sciences, Department of Medical Oncology, New Delhi, India

**Keywords:** Myocardial infarction after rituximab

## TO THE EDITOR

Rituximab (anti-CD20) is a chimeric monoclonal antibody and is commonly used in treatment of various lymphomas and nonmalignant immune disorders. Infusion reactions are common following rituximab administration. Cardiotoxicity with rituximab is less frequent, but occasionally arrhythmia is reported. We report here an acute myocardial infarction following the first rituximab infusion for the treatment of splenic lymphoma with villous lymphocytes (SLVL).

A previously healthy 65-year-old male presented to our hospital in May 2013 with a 6-month history of fatigue and a feeling of fullness over the left side of his abdomen. Clinical examination revealed ECOG performance status of 1 at the time of first visit, mild pallor, and massive splenomegaly (12 cm below the costal margin). His hemogram showed a hemoglobin level of 10.5 g/dL, total leukocyte count of 3.4x10^9^/L, and platelet count of 50x10^9^/L. Peripheral blood smear examination showed normochromic normocytic anemia and leucopenia with normal differential count. Renal and liver functions were within normal limits. Coomb’s test results were negative and there were no features of hemolysis. Electrocardiogram and 2D echo results were normal. Further diagnostic and staging workup including bone marrow biopsy, touch, and imaging revealed stage IV splenic lymphoma with villous lymphocytes. It was planned to start BR (bendamustine and rituximab) combination chemo/immunotherapy, in which rituximab was the initial therapy. Rituximab was administered as per standard prescribing recommendations with acetaminophen, diphenhydramine, and steroids. The patient complained of uneasiness and sweating for a few minutes after 5 min of controlled infusion. Electrocardiogram revealed ST elevation in inferior leads, which did not subside even after stopping the rituximab infusion. The troponin T level was elevated. The patient was shifted to the coronary care unit (CCU) and angiography showed a complete thrombotic occlusion of the right coronary artery, in the absence of abnormalities in the other coronary arteries. Angioplasty was done and he was discharged from the CCU after 2 days in stable condition. 

Rituximab is generally well tolerated if given with adequate premedication. Grade 1/2 infusion-related drug reactions are well known, which include fever, chills, hypotension, and dyspnea; pretreatment with acetaminophen, antihistamines, and, on occasion, corticosteroids minimizes these symptoms. Cardiovascular toxicities described with rituximab include tachycardia, hypotension, hypertension, hypoxemia, arrhythmias, bradycardia, and atrial fibrillation. Myocardial infarction as a complication of rituximab infusion is listed in the package insert but is not clearly described in the literature [1]. Myocardial infarction after rituximab infusion is extremely rare and was described in only 4 cases of lymphoid malignancy (Table 1) and 2 cases of immune-mediated disorders [2,3,4]. The proposed mechanism for Acute Coronary syndrome following rituximab infusion is the release of cytokines, which cause vasoconstriction, platelet activation, and/or rupture of atherosclerotic plaque [5]. In our case it is likely that the patient had preexisting vulnerable plaque in light of his advanced age as a risk factor. Meticulous screening for ischemic heart disease may be necessary in high-risk subsets before starting rituximab therapy. Awareness of this complication is important to minimize the risk of treatment-related morbidity.

## CONFLICT OF INTEREST STATEMENT

The authors of this paper have no conflicts of interest, including specific financial interests, relationships, and/ or affiliations relevant to the subject matter or materials included.

## Figures and Tables

**Table 1:**
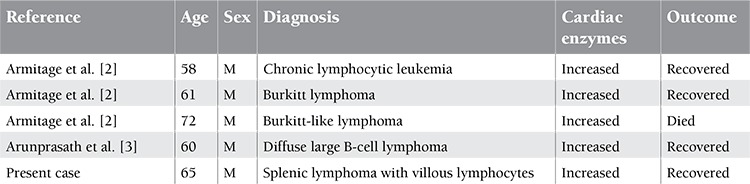
Patients with acute myocardial infarction after first infusion of rituximab in lymphoid malignancies.
